# Supplementary material for: Dynalign II: common secondary structure prediction for RNA homologs with domain insertions
Source: Nucleic Acids Res. 2014 Nov 21;42(22):13939–48. doi: 10.1093/nar/gku1172 (PMC4267632; doi:10.1093/nar/gku1172)
Supplement: SUPPLEMENTARY DATA [file supp_gku1172_nar-02021-z-2014-File012.zip › manual/javadoc/RNAstructure_java_interface/source/windows/DrawingWindow.html]

DrawingWindow


JavaScript is disabled on your browser.


- Overview
- Package
- Class
- Tree
- Index
- Help

- Prev Class
- Next Class

- Frames
- No Frames

- All Classes

- Summary:
- Nested |
- Field |
- Constr |
- Method

- Detail:
- Field |
- Constr |
- Method


RNAstructure\_java\_interface.source.windows

## Class DrawingWindow

- java.lang.Object
- - java.awt.Component
  - - java.awt.Container
    - - javax.swing.JComponent
      - - javax.swing.JInternalFrame
        - - RNAstructure\_java\_interface.source.windows.InternalWindow
          - - RNAstructure\_java\_interface.source.windows.DrawingWindow

- All Implemented Interfaces:
  :   ActionListener, ImageObserver, MenuContainer, Serializable, EventListener, Accessible, RootPaneContainer, WindowConstants

  ---

    

  ```
  public class DrawingWindow
  extends InternalWindow
  ```

  A class responsible for displaying a drawing window.
    
    
  This class is a wrapper for the self-contained drawing framework dialogs,
  which essentially converts them into internal frames but does not take over
  or duplicate any of their functions.
    
    
  Most drawings, menus, or listeners are transplanted from the drawing
  framework dialogs.

  See Also:
  :   Serialized Form

- - ### Nested Class Summary

    - ### Nested classes/interfaces inherited from class javax.swing.JInternalFrame

      `JInternalFrame.AccessibleJInternalFrame, JInternalFrame.JDesktopIcon`
    - ### Nested classes/interfaces inherited from class javax.swing.JComponent

      `JComponent.AccessibleJComponent`
    - ### Nested classes/interfaces inherited from class java.awt.Container

      `Container.AccessibleAWTContainer`
    - ### Nested classes/interfaces inherited from class java.awt.Component

      `Component.AccessibleAWTComponent, Component.BaselineResizeBehavior, Component.BltBufferStrategy, Component.FlipBufferStrategy`
  - ### Field Summary

    Fields

    | Modifier and Type | Field and Description |
    | --- | --- |
    | `private ImageDialog` | `dialog` The image dialog connected to this window. |
    | `private boolean` | `error` True if an error occurred building this window, false if not. |
    | `private static long` | `serialVersionUID` |
    | `private int` | `strand` The strand being drawn. |

    - ### Fields inherited from class RNAstructure\_java\_interface.source.windows.InternalWindow

      `backend, dialogHandler, menuBar`
    - ### Fields inherited from class javax.swing.JInternalFrame

      `closable, CONTENT_PANE_PROPERTY, desktopIcon, FRAME_ICON_PROPERTY, frameIcon, GLASS_PANE_PROPERTY, iconable, IS_CLOSED_PROPERTY, IS_ICON_PROPERTY, IS_MAXIMUM_PROPERTY, IS_SELECTED_PROPERTY, isClosed, isIcon, isMaximum, isSelected, LAYERED_PANE_PROPERTY, maximizable, MENU_BAR_PROPERTY, resizable, ROOT_PANE_PROPERTY, rootPane, rootPaneCheckingEnabled, title, TITLE_PROPERTY`
    - ### Fields inherited from class javax.swing.JComponent

      `accessibleContext, listenerList, TOOL_TIP_TEXT_KEY, ui, UNDEFINED_CONDITION, WHEN_ANCESTOR_OF_FOCUSED_COMPONENT, WHEN_FOCUSED, WHEN_IN_FOCUSED_WINDOW`
    - ### Fields inherited from class java.awt.Component

      `BOTTOM_ALIGNMENT, CENTER_ALIGNMENT, LEFT_ALIGNMENT, RIGHT_ALIGNMENT, TOP_ALIGNMENT`
    - ### Fields inherited from interface javax.swing.WindowConstants

      `DISPOSE_ON_CLOSE, DO_NOTHING_ON_CLOSE, EXIT_ON_CLOSE, HIDE_ON_CLOSE`
    - ### Fields inherited from interface java.awt.image.ImageObserver

      `ABORT, ALLBITS, ERROR, FRAMEBITS, HEIGHT, PROPERTIES, SOMEBITS, WIDTH`
  - ### Constructor Summary

    Constructors

    Constructor and Description

    `DrawingWindow(String file)`

    File Constructor.

    `DrawingWindow(String file,
    int strand)`

    Strand Constructor.
  - ### Method Summary

    Methods

    | Modifier and Type | Method and Description |
    | --- | --- |
    | `protected void` | `doActions(String command)` Do any actions specific to this window. |
    | `private void` | `doRevalidate()` Revalidate the drawing window. |
    | `private void` | `getImageData()` Get the data from a dialog to put in this window. |
    | `boolean` | `isError()` Get whether this window encountered an error. |
    | `protected RolloverMenu[]` | `setMenus()` Create the array of variable menus used by this window. |

    - ### Methods inherited from class RNAstructure\_java\_interface.source.windows.InternalWindow

      `actionPerformed, setTitles, viewWindow`
    - ### Methods inherited from class javax.swing.JInternalFrame

      `addImpl, addInternalFrameListener, createRootPane, dispose, doDefaultCloseAction, fireInternalFrameEvent, getAccessibleContext, getContentPane, getDefaultCloseOperation, getDesktopIcon, getDesktopPane, getFocusCycleRootAncestor, getFocusOwner, getFrameIcon, getGlassPane, getInternalFrameListeners, getJMenuBar, getLastCursor, getLayer, getLayeredPane, getMenuBar, getMostRecentFocusOwner, getNormalBounds, getRootPane, getTitle, getUI, getUIClassID, getWarningString, hide, isClosable, isClosed, isFocusCycleRoot, isIcon, isIconifiable, isMaximizable, isMaximum, isResizable, isRootPaneCheckingEnabled, isSelected, moveToBack, moveToFront, pack, paintComponent, paramString, remove, removeInternalFrameListener, reshape, restoreSubcomponentFocus, setClosable, setClosed, setContentPane, setCursor, setDefaultCloseOperation, setDesktopIcon, setFocusCycleRoot, setFrameIcon, setGlassPane, setIcon, setIconifiable, setJMenuBar, setLayer, setLayer, setLayeredPane, setLayout, setMaximizable, setMaximum, setMenuBar, setNormalBounds, setResizable, setRootPane, setRootPaneCheckingEnabled, setSelected, setTitle, setUI, show, toBack, toFront, updateUI`
    - ### Methods inherited from class javax.swing.JComponent

      `addAncestorListener, addNotify, addVetoableChangeListener, computeVisibleRect, contains, createToolTip, disable, enable, firePropertyChange, firePropertyChange, firePropertyChange, fireVetoableChange, getActionForKeyStroke, getActionMap, getAlignmentX, getAlignmentY, getAncestorListeners, getAutoscrolls, getBaseline, getBaselineResizeBehavior, getBorder, getBounds, getClientProperty, getComponentGraphics, getComponentPopupMenu, getConditionForKeyStroke, getDebugGraphicsOptions, getDefaultLocale, getFontMetrics, getGraphics, getHeight, getInheritsPopupMenu, getInputMap, getInputMap, getInputVerifier, getInsets, getInsets, getListeners, getLocation, getMaximumSize, getMinimumSize, getNextFocusableComponent, getPopupLocation, getPreferredSize, getRegisteredKeyStrokes, getSize, getToolTipLocation, getToolTipText, getToolTipText, getTopLevelAncestor, getTransferHandler, getVerifyInputWhenFocusTarget, getVetoableChangeListeners, getVisibleRect, getWidth, getX, getY, grabFocus, isDoubleBuffered, isLightweightComponent, isManagingFocus, isOpaque, isOptimizedDrawingEnabled, isPaintingForPrint, isPaintingOrigin, isPaintingTile, isRequestFocusEnabled, isValidateRoot, paint, paintBorder, paintChildren, paintImmediately, paintImmediately, print, printAll, printBorder, printChildren, printComponent, processComponentKeyEvent, processKeyBinding, processKeyEvent, processMouseEvent, processMouseMotionEvent, putClientProperty, registerKeyboardAction, registerKeyboardAction, removeAncestorListener, removeNotify, removeVetoableChangeListener, repaint, repaint, requestDefaultFocus, requestFocus, requestFocus, requestFocusInWindow, requestFocusInWindow, resetKeyboardActions, revalidate, scrollRectToVisible, setActionMap, setAlignmentX, setAlignmentY, setAutoscrolls, setBackground, setBorder, setComponentPopupMenu, setDebugGraphicsOptions, setDefaultLocale, setDoubleBuffered, setEnabled, setFocusTraversalKeys, setFont, setForeground, setInheritsPopupMenu, setInputMap, setInputVerifier, setMaximumSize, setMinimumSize, setNextFocusableComponent, setOpaque, setPreferredSize, setRequestFocusEnabled, setToolTipText, setTransferHandler, setUI, setVerifyInputWhenFocusTarget, setVisible, unregisterKeyboardAction, update`
    - ### Methods inherited from class java.awt.Container

      `add, add, add, add, add, addContainerListener, addPropertyChangeListener, addPropertyChangeListener, applyComponentOrientation, areFocusTraversalKeysSet, countComponents, deliverEvent, doLayout, findComponentAt, findComponentAt, getComponent, getComponentAt, getComponentAt, getComponentCount, getComponents, getComponentZOrder, getContainerListeners, getFocusTraversalKeys, getFocusTraversalPolicy, getLayout, getMousePosition, insets, invalidate, isAncestorOf, isFocusCycleRoot, isFocusTraversalPolicyProvider, isFocusTraversalPolicySet, layout, list, list, locate, minimumSize, paintComponents, preferredSize, printComponents, processContainerEvent, processEvent, remove, removeAll, removeContainerListener, setComponentZOrder, setFocusTraversalPolicy, setFocusTraversalPolicyProvider, transferFocusDownCycle, validate, validateTree`
    - ### Methods inherited from class java.awt.Component

      `action, add, addComponentListener, addFocusListener, addHierarchyBoundsListener, addHierarchyListener, addInputMethodListener, addKeyListener, addMouseListener, addMouseMotionListener, addMouseWheelListener, bounds, checkImage, checkImage, coalesceEvents, contains, createImage, createImage, createVolatileImage, createVolatileImage, disableEvents, dispatchEvent, enable, enableEvents, enableInputMethods, firePropertyChange, firePropertyChange, firePropertyChange, firePropertyChange, firePropertyChange, firePropertyChange, getBackground, getBounds, getColorModel, getComponentListeners, getComponentOrientation, getCursor, getDropTarget, getFocusListeners, getFocusTraversalKeysEnabled, getFont, getForeground, getGraphicsConfiguration, getHierarchyBoundsListeners, getHierarchyListeners, getIgnoreRepaint, getInputContext, getInputMethodListeners, getInputMethodRequests, getKeyListeners, getLocale, getLocation, getLocationOnScreen, getMouseListeners, getMouseMotionListeners, getMousePosition, getMouseWheelListeners, getName, getParent, getPeer, getPropertyChangeListeners, getPropertyChangeListeners, getSize, getToolkit, getTreeLock, gotFocus, handleEvent, hasFocus, imageUpdate, inside, isBackgroundSet, isCursorSet, isDisplayable, isEnabled, isFocusable, isFocusOwner, isFocusTraversable, isFontSet, isForegroundSet, isLightweight, isMaximumSizeSet, isMinimumSizeSet, isPreferredSizeSet, isShowing, isValid, isVisible, keyDown, keyUp, list, list, list, location, lostFocus, mouseDown, mouseDrag, mouseEnter, mouseExit, mouseMove, mouseUp, move, nextFocus, paintAll, postEvent, prepareImage, prepareImage, processComponentEvent, processFocusEvent, processHierarchyBoundsEvent, processHierarchyEvent, processInputMethodEvent, processMouseWheelEvent, remove, removeComponentListener, removeFocusListener, removeHierarchyBoundsListener, removeHierarchyListener, removeInputMethodListener, removeKeyListener, removeMouseListener, removeMouseMotionListener, removeMouseWheelListener, removePropertyChangeListener, removePropertyChangeListener, repaint, repaint, repaint, resize, resize, setBounds, setBounds, setComponentOrientation, setDropTarget, setFocusable, setFocusTraversalKeysEnabled, setIgnoreRepaint, setLocale, setLocation, setLocation, setName, setSize, setSize, show, size, toString, transferFocus, transferFocusBackward, transferFocusUpCycle`
    - ### Methods inherited from class java.lang.Object

      `clone, equals, finalize, getClass, hashCode, notify, notifyAll, wait, wait, wait`

- - ### Field Detail


    - #### dialog

      ```
      private ImageDialog dialog
      ```

      The image dialog connected to this window.


    - #### error

      ```
      private boolean error
      ```

      True if an error occurred building this window, false if not.


    - #### serialVersionUID

      ```
      private static final long serialVersionUID
      ```

      See Also:
      :   Constant Field Values


    - #### strand

      ```
      private int strand
      ```

      The strand being drawn.
  - ### Constructor Detail


    - #### DrawingWindow

      ```
      public DrawingWindow(String file)
      ```

      File Constructor.

      Parameters:
      :   `file` - The file whose data should be drawn on the window.


    - #### DrawingWindow

      ```
      public DrawingWindow(String file,
                   int strand)
      ```

      Strand Constructor.

      Parameters:
      :   `file` - The dot plot data file to draw.
      :   `strand` - The strand from the data file to draw.
  - ### Method Detail


    - #### doActions

      ```
      protected void doActions(String command)
      ```

      Do any actions specific to this window.
        
        
      Note that most of the actions for this window are handled by the menus
      in the self-contained drawing framework.

      **Specified by:**
      :   `doActions` in class `InternalWindow`

      Parameters:
      :   `command` - The command that signifies a particular action.


    - #### doRevalidate

      ```
      private void doRevalidate()
      ```

      Revalidate the drawing window.


    - #### getImageData

      ```
      private void getImageData()
      ```

      Get the data from a dialog to put in this window.


    - #### isError

      ```
      public boolean isError()
      ```

      Get whether this window encountered an error.

      Returns:
      :   True if an error occurred, false if not.


    - #### setMenus

      ```
      protected RolloverMenu[] setMenus()
      ```

      Create the array of variable menus used by this window.
        
        
      Note that this version of the function does nothing, since this window
      is only a wrapper for a self-contained drawing dialog.
        
        
      Menus are added dynamically in the drawing window class, because this
      class can hold multiple types of drawings with different menus.

      **Specified by:**
      :   `setMenus` in class `InternalWindow`

      Returns:
      :   The array of variable menus created for this window.


- Overview
- Package
- Class
- Tree
- Index
- Help

- Prev Class
- Next Class

- Frames
- No Frames

- All Classes

- Summary:
- Nested |
- Field |
- Constr |
- Method

- Detail:
- Field |
- Constr |
- Method
